# Alginate-Collagen Fibril Composite Hydrogel

**DOI:** 10.3390/ma8020799

**Published:** 2015-02-16

**Authors:** Mahmoud Baniasadi, Majid Minary-Jolandan

**Affiliations:** Department of Mechanical Engineering, University of Texas at Dallas, 800 W. Campbell Rd, Richardson, TX 75080, USA; E-Mail: Mahmoud.Baniasadi@utdallas.edu

**Keywords:** composite hydrogel, alginate, type I collagen fibril, mechanical properties, AFM nanoindentation, tensile test, rheometry

## Abstract

We report on the synthesis and the mechanical characterization of an alginate-collagen fibril composite hydrogel. Native type I collagen fibrils were used to synthesize the fibrous composite hydrogel. We characterized the mechanical properties of the fabricated fibrous hydrogel using tensile testing; rheometry and atomic force microscope (AFM)-based nanoindentation experiments. The results show that addition of type I collagen fibrils improves the rheological and indentation properties of the hydrogel.

## 1. Introduction

Hydrogels are being extensively used for tissue engineering applications. There has been a remarkable effort in producing properties of hydrogels as close to the native tissue microenvironment as possible [1,2]. The properties of interest include physical properties, biochemical properties, and biological properties. For example, most tissues in the human body are hierarchically structured, involving length-scales from nanoscale to macroscale [3,4]. Accordingly, one of the major routes in making more biomimetic hydrogels involves adding micro/nanostructures to the host hydrogel polymer [5]. These nanostructures include polymeric, inorganic/ceramic and metallic nanoparticles, nanotubes, nanowires, graphene, nanodiamonds, *etc.* [6,7,8,9,10,11,12,13,14,15,16,17]. The components often add extra functionalities to the based hydrogel polymer, such as electrical, physical, chemical, and biological functionalities. It is desirable for the added components to impart these functionalities with minimal or no compromise to the other original properties of the host hydrogel. The obtained hydrogel is often termed a nanocomposite or hybrid hydrogel [15,18,19,20,21,22,23]. A recent review summarized the latest progress in nanocomposite hydrogels [24].

Nanofiber-reinforced hydrogels are a class of nanocomposite hydrogels, in which often electrospun polymeric nanofibers are added to the hydrogel matrix [25,26,27,28,29,30,31,32,33]. Recent work reported on transparent electrospun gelatin nanofibers infiltrated with alginate hydrogel for cornea tissue engineering [25]. Addition of nanofibers enhanced the elastic modulus of the hydrogel by several folds. In another study, 3D rapid prototyping technique was used to form a crossed log-pile of elastic fibers that were subsequently impregnated with an epoxy-based hydrogel [32].

**Figure 1:**
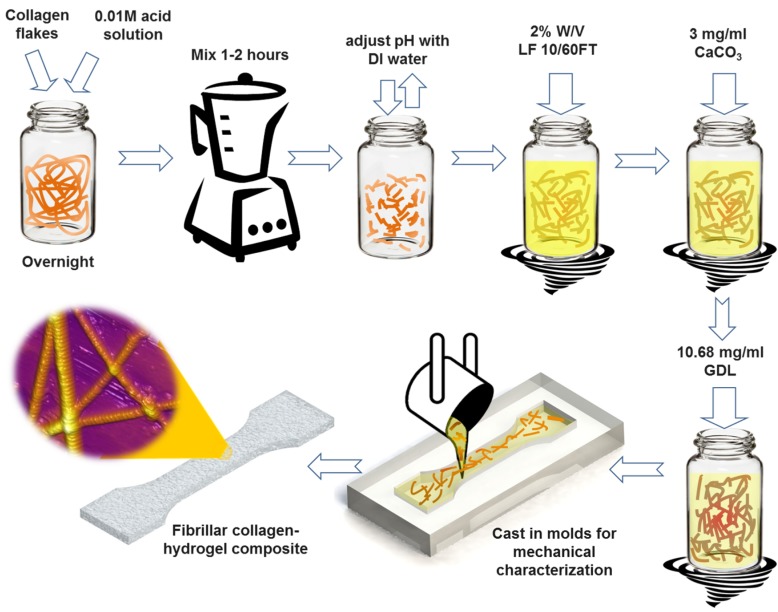
Schematic of the step-by-step preparation process of hybrid fibrous composite hydrogel with incorporation of native type I collagen fibrils. LF 10/60FT is a sodium alginate from FMC Biopolymer (Philadelphia, PA, USA), GDL is d-(+)-Gloconic acid δ-lactone. See Experimental Section.

In this article, we report on the synthesis and characterization of a composite fibrous hydrogel by incorporation of native type I collagen fibrils into the alginate hydrogel. The process for the synthesis of the collagen-alginate composite hydrogel is schematically shown in Figure 1. Briefly, alginate was added to solutions of collagen fibrils of various concentrations. The resulting mixture was subsequently cross-linked by calcium ions using calcium carbonate (CaCO_3_).

Alginate hydrogel is being widely used for cell encapsulation, cell transplantation, drug delivery, and tissue engineering applications [34,35,36]. Although alginate possesses many favorable properties for tissue engineering applications, it lacks specific interaction with mammalian cells. Therefore, it is often functionalized with RGD-containing cell adhesion ligands [35]. Tripeptide Arg-Gly-Asp (RGD) is a common cell-recognition ligand in extra cellular matrix that binds integrins to the membrane proteins of different cell types. This cell adhesion ligand is present in collagen fibrils in tissues. Accordingly, composite hydrogel of alginate-collagen may provide the adhesion sites for cell adhesion. In addition, alginate hydrogel lacks the hierarchical fibrous structure of native tissues. Nanofibers such as electrospun nanofibers can be added to alginate hydrogel to provide the hierarchical structure of the native tissues. Native collagen has several advantages in this regard. It adds fibrous structure to the alginate hydrogel. In addition, it provides the RGD-binding sites for the cell adhesion. Finally, collagen possesses the characteristic nano-topography feature, in contrast to the smooth surface of synthetic nanofibers.

**Figure 2:**
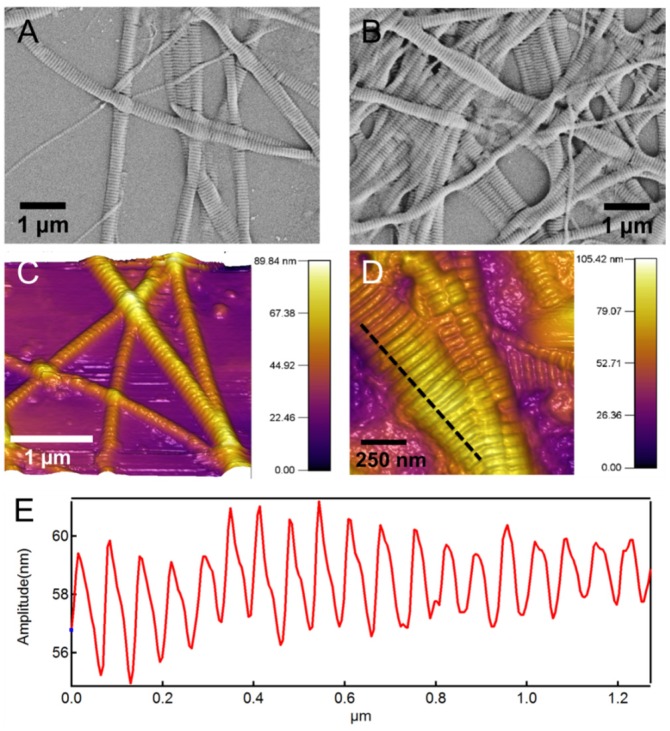
(**A**,**B**) Scanning electron microscope (SEM) morphology and (**C**,**D**) atomic force microscope (AFM) topography images of the native type I collagen fibrils used for synthesis of the composite hydrogel. The characteristic periodic pattern of 60–70 nm is apparent in images; (**E**) Shows a line-profile taken along the dashed line in (**D**) and shows this periodic pattern.

Collagen fibrils are major components of extracellular matrix (ECM) and connectives tissues such as bone and tendon, as well as in tissues such as cornea. Similar to alginate, collagen hydrogels have been used for various applications including investigation of adherence of bone marrow stromal cells, as scaffold for cartilage tissue engineering, in vascular grants, and for applications in wound healing and as a pro-angiogenic site for islet transplantation [37,38,39,40,41]. Native type I collagen fibrils have characteristic periodic patterns of 60–70 nm [42] (See Figure 2). In addition to the RGD binding sites, these highly periodic nano-topographical features are believed to be important for cell adhesion and growth [39,43,44,45,46,47]. The majority of synthetic nanofibers including electrospun nanofibers that are being used in scaffold for tissue engineering lack this important nano-topographical feature.

Figure 2 shows scanning electron microscope (SEM) and atomic force microscope (AFM) images of the native collagen fibrils. In both images the characteristic periodic banding of collagen is apparent. The subset in Figure 2D shows a line-profile taken along the dashed line that more clearly shows this periodic banding pattern with a periodicity of 60–70 nm. This periodic pattern arises from the special microstructural arrangement of collagen molecules in a “quarter stagger” arrangement [42,48]. The periodicity is 60–70 nm, which results in the so-called “gap” and “overlap” regions. The diameter of the individual collagen fibrils varies from 50 to 200 nm based on our previous study [42].

Collagen hydrogels are often prepared from collagen molecules (or triple helix structure). However, type I collagen in native tissue is fibrillar with a characteristic periodic pattern. The *in vitro* fibrillogenesis of collagen triple helix to fibril is performed by adjusting the pH from the original acidic solution to pH ~7.4, a process that is still not fully understood. Although collagen in this neutralization process self-assembles to a filamentous structure, however, the *in vitro* assembled collagen fibril may lack the characteristic banding pattern (~67 nm) of native collagen [49]. It has been shown than some of the assembled collagen in the fibrillogenesis process includes both fibrils that display a periodic banding pattern and filamentous structures that do not have this characteristic collagen striation [49].

The elastic moduli of alginate hydrogels vary considerably depending on gelling conditions and cross-linking [36]. The most common cross-linking is ionic cross-linking by Ca^2+^ ions [50], although photocross-linked alginate hydrogels have been also reported [51]. Elastic moduli of ionically cross-linked (CaCO_3_) alginate hydrogels measured using compression experiments were reported to be from 5 to 120 kPa, depending on the concentration of the Ca^2+^ ions, which was varied from 0.5% to 5% [50]. The compression modulus of photocross-linked hydrogel was reported to be ~170 kPa [51]. Mechanical properties of collagen hydrogel covalently functionalized with three different monomers, *i.e.*, 4-vinylbenzyl chloride, glycidyl methacrylate and methacrylic anhydride were characterized using atomic force microscope (AFM) [52]. By adjusting the degree of functionalization, an elastic modulus in the range of 16–387 kPa was obtained. Similar to other reports, no collagen fibril formation was observed in these specimens [52]. A biomimetic fibrillar collagen scaffold was recently introduced. By altering the freeze drying conditions through introduction of multiple temperature gradients, collagen scaffolds with complex pore orientations, and anisotropy in pore size and alignment were produced. However, no mechanical properties were reported [53]. The mechanical properties of collagen-chitosan hydrogel were characterized using compression experiment [41]. For this hydrogel, soluble collagen molecules were used after pH adjustment, which resulted in fibril formation. The elastic modulus of the pure collagen hydrogel was measured to be 0.4 kPa as compared to 0.7 kPa for collagen-chitosan hydrogel [41].

Although mechanical properties of alginate hydrogel and collagen hydrogel have been reported in the literature, the properties of the composite hydrogel are not available. In addition, for composite hydrogel of this type, mechanical properties depend on the type of loading and deformation, given their fibrous and heterogeneous microstructure. Therefore, the behavior of the composite hydrogel in shear, tension, and indentation could be different. We used rheometry (shear deformation), tensile test and atomic force microscopy (AFM)-based nanoindentation to characterize the mechanical properties of the fabricated hydrogel samples.

## 2. Results and Discussion

**Figure 3:**
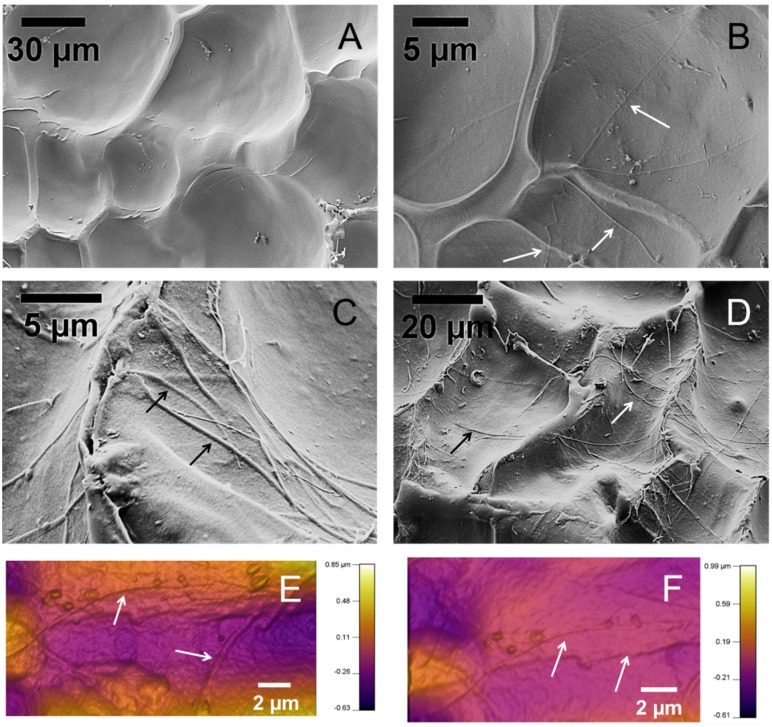
SEM and AFM images of the surface of the hydrogel samples. (**A**) A hydrogel sample with no collagen fibrils; (**B**–**D**) Composite hydrogel samples with collagen fibrils, (**B**) 1X, (**C**,**D**) 2X; (**E**,**F**) AFM topography images of collagen-alginate hydrogel samples. Arrows point to collagen fibrils.

Figure 3 shows representative SEM images of the surface of the hydrogel samples. The samples were freeze-dried for observation in SEM. Figure 3A is the hydrogel sample without collagen fibrils. Figure 3B–D are SEM images of samples with collagen fibrils. Collagen fibrils are apparent on the surface of the specimens, pointed to by arrows. Collagen fibrils are several microns long and several hundred in diameter, similar to the isolated collagen fibrils shown in Figure 2. Several additional SEM images of the hydrogels are presented in Figure S1.

**Figure 4:**
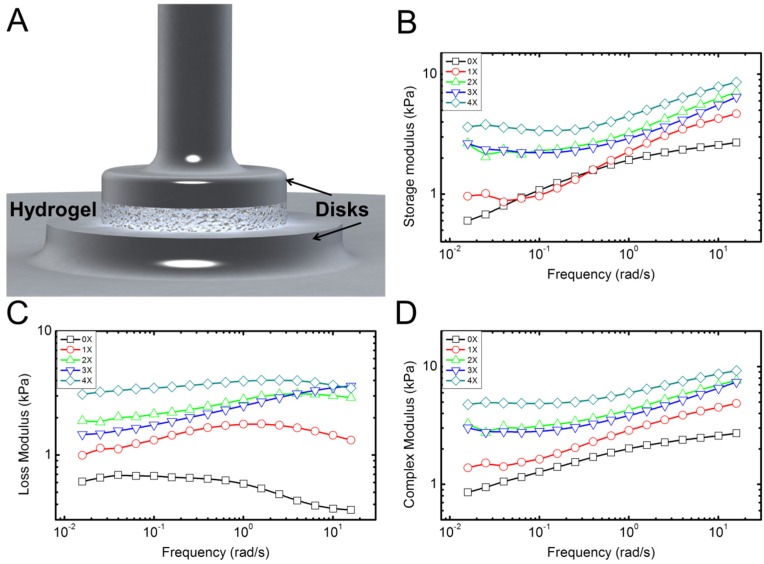
Characterization of the rheological properties of the hydrogel samples. (**A**) Schematic shows the disk-shaped hydrogel sample between the two circular disks of the rheometer; (**B**–**D**) Typical plots of the storage, loss, and complex moduli, respectively, extracted from the rheological measurements. Five samples for each concentration were tested.

Typical experimental results for rheological properties of the hydrogel samples with different concentration of collagen fibrils are shown in Figure 4. These display typical behavior for the five samples tested for each concentration. Overall, the behavior was consistent with less than 9% error between different samples. Rheometery measures mechanical properties of the samples in terms of storage (*E*') and loss moduli (*E*'') under combined compressive and shear deformations. Storage modulus represents the elastic energy, while the loss modulus measures the dissipative energy. The storage modulus of hydrogel with no collagen fibril (alginate hydrogel) content increases by increasing the frequency (deformation rate), as shown in Figure 4B. Addition of collagen preserves this trend. The loss modulus of hydrogel with no collagen content is initially constant and then decreases by increasing the frequency. This means that energy dissipation, which is related to the toughness of the material decreases with frequency. However, addition of collagen appears to reverse this trend. Data in Figure 4C suggest that this increase in loss modulus is more apparent for higher frequencies. In Figure 4B, the data for 0X and 1X specimens show overlap for low frequency up to 1 Hz. From 1 to 10 Hz, the 1X specimen shows larger storage modulus. The loss modulus, however, shows larger values for 1X in all frequencies. There could be a possible explanations for overlap between 0X and 1X in Figure 4B as follows: It seems that contribution of collagen to the storage modulus starts at high frequencies >1 Hz, while for 0.01 Hz to ~1 HZ, the contribution of collagen to the storage modulus is negligible. However, the contribution of collagen to the loss modulus occurs for all frequencies from 0.01 to 10 Hz. It is possible that for low concentration of 1X and in small frequencies, collagen fibrils slide within the alginate matrix. As such they will not contribute to the elastic properties; however, the sliding deformation will cause energy dissipation appearing in the loss modulus. As the frequency increases, the sliding of collagen samples may reduce, which results in enhancement of the storage modulus and decrease in the loss modulus, as shown in Figure 4C. In addition, the data for 2X and 3X concentrations appear to overlap for all frequencies. Collagen fibrils in our study have a very low concentration. Based on SEM images shown in Figure 2 and Figure S1, the distribution of collagen in samples is not fully homogenous at different points of the sample. We believe that the overlaps in data could be an error introduced by random orientation and dispersion of collagen fibrils in the specimens. Statistical analysis (Two way ANOVA, *p* <0.05) followed with a Tukey test clearly confirmed significant changes in storage and loss moduli—and as a result, complex modulus increased. Overall, addition of small quantities of collagen fibrils results in a several times increase in elastic and loss moduli of the hydrogel samples, which is apparent in the results of the complex modulus (*E*' + *iE*'') in Figure 4D.

**Figure 5:**
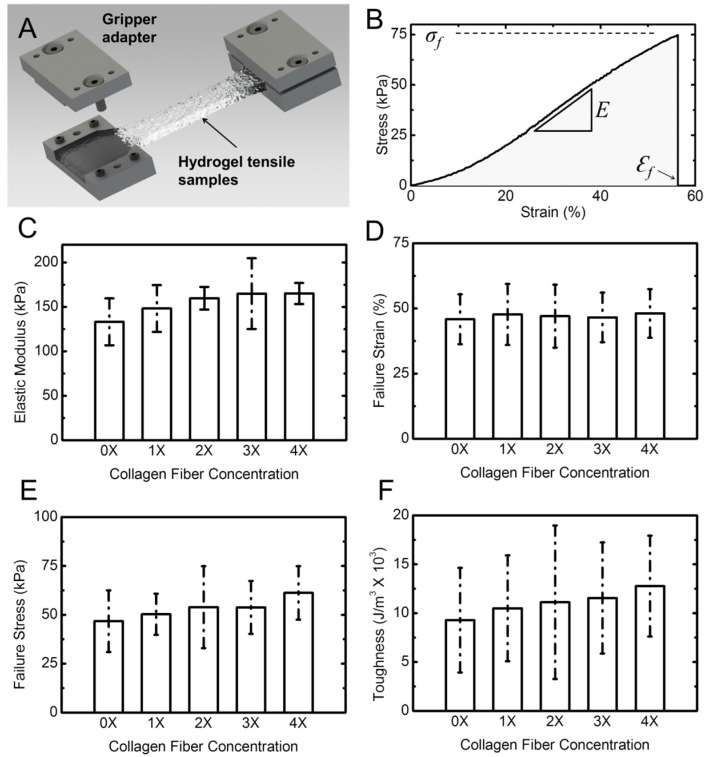
Tensile properties of hydrogel samples. (**A**) Schematic shows the dog-bone shaped hydrogel sample gripped using costume-designed gripper adaptor; (**B**) A typical stress-strain response from a hydrogel sample. The slope of the linear section of the response was used to extract the elastic (Young’s) modulus of the hydrogel specimen; (**C**) The Young’s modulus; (**D**) failure strain; (**E**) failure stress; and (**F**) toughness of hydrogel *vs.* collagen concentration. Error bars represent standard deviation (SD) for five samples for each concentration.

Figure 5A shows a schematic of the tensile specimens in a costume-designed gripper adapter. A typical stress-strain response of the hydrogel samples is shown in Figure 5B. The response is presented in terms of engineering stress *vs.* engineering strain. Stress was obtained by dividing the force by the initial cross-sectional area of the sample. The cross-section was measured using a caliper at three different points in the gauge length of the sample and the average value was used. Strain was calculated by dividing the cross-head displacement by the original length of the sample. Stress–strain response of the samples shows a typical J-response (nonlinear) initially followed by a linear response. We used the slope of the linear section for each of the samples to obtain the elastic (Young’s) modulus [34,54]. In addition, for each experiment we obtained the failure strain, the failure stress (strength), and the toughness. The toughness was calculated from the area under the stress–strain response, as shown with the shaded area in Figure 5B. From statistical analysis (Two way ANOVA, *p* < 0.05) followed with a Tukey test of tensile results, it can be concluded that, in contrast to rheological properties, addition of collagen fibril has minimal effect on the tensile properties of the composite hydrogel. There is a minimal increasing trend for elastic modulus, failure stress, and toughness. However, the failure strain does not change by addition of collagen fibrils. Table S1 gives detailed tensile properties of the tested samples. It should be noted that collagen fibrils are randomly distributed in the hydrogel samples, and are not necessarily aligned with the tension direction. With future improvement for aligning the fibrils, the tensile properties may show larger improvement.

**Figure 6:**
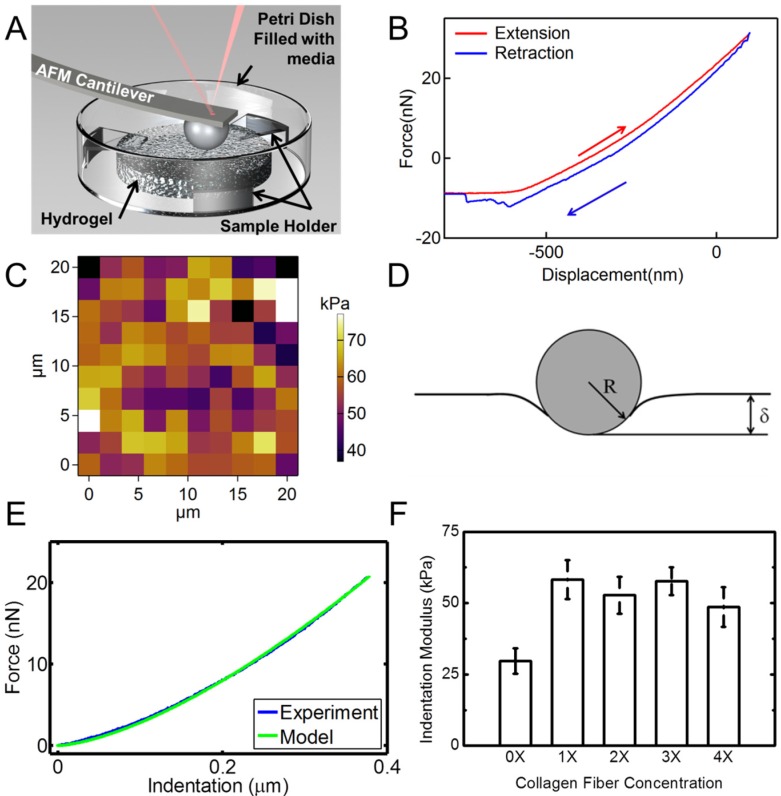
AFM nanoindentation experiments on hydrogel specimens. (**A**) Schematic shows the setup for indentation of hydrogel samples in a liquid media using a spherical probe; (**B**) A typical force-displacement (indentation depth) response from hydrogel; (**C**) Indentation map on a 20 µm × 20 µm area for a total of 100 points of a sample with 4X collagen concentration. The color bar represents the indentation modulus; (**D**) Schematic of the Hertzian contact mechanics model for spherical indentation into the hydrogel surface; (**E**) Comparison of the force-indentation data from an experiment modeled with Hertzian contact mechanics; (**F**) Indentation moduli extracted from nanoindentation experiments for hydrogel samples with different concentrations of collagen fibrils. Error bars in (**F)** are SD for five measurements at each concentration.

Figure 6 shows the results of the nanoindentation experiment. Hydrogel samples were indented using an AFM probe with a spherical tip inside a liquid medium as schematically shown in Figure 6A. Figure 6B is a typical extension-retraction response from an indentation experiment. For each sample, a map of 100 indentation points was obtained. A typical map is shown in Figure 6C. Several additional indentation maps are given in Figure S2. The color code represents the indentation modulus with the bright colors showing larger indentation modulus values.

We modeled the nanoindentation of the spherical tip into the hydrogel using the Hertzian contact mechanics model to derive the indentation modulus of the sample, Figure 6D [55]. Based on the Hertzian model, for indentation of an elastic half-space using a spherical indenter, the indentation load-displacement relation is given as [56]:

(1) $$F=\frac{4}{3}E_{r}\;R^{1/2}\delta^{3/2}$$

In this equation, δ is the deformation of the samples in contact, shown in Figure 6D. *F* is the indentation force, *E_r_* is the reduced elastic modulus of the tip and the sample, and *R* is the tip radius [57,58]. The reduced elastic modulus is given as:

(2) $$\frac{1}{E_{r}}=\frac{\left(1-\upsilon_{t}^{2}\right)}{E_{t}}+\frac{\left(1-\upsilon_{s}^{2}\right)}{E_{s}}$$

where “*t*” and “*s*” represent tip and sample, respectively. We note that since the hydrogel is much softer than the SiO_2_ (silicone dioxide) spherical probe, the deformation of the probe tip is negligible. In this case, the reduced elastic modulus can be replaced with the elastic modulus of the hydrogel. This model was introduced into a MATLAB code. Each force-indentation depth curve was modeled using this model. A typical fitted model is shown in Figure 6E. The Poisson’s ratio of the sample was assumed to be ~0.49, which is a common assumption for hydrated materials, given the incompressible nature of water. Hence, the indentation modulus was the only fitting parameter. We conducted indentation experiments with displacement rates of 0.5–3 µm/s to ensure that the indentation rate does not have any effect on the results in this displacement range. The results for different rates in this range showed similar behavior (Figure S3). Indentation modulus *vs.* fibril concentration is shown in Figure 6F. These data are the average of different indentation rates. As observed from statistical analysis (Two way ANOVA, *p* < 0.05) followed with a Scheffe test, the addition of collagen results in a significant increase of the indentation modulus of the hydrogel samples. Data for various collagen concentrations do not show significant variations. This may be explained by considering the random orientation and dispersion of collagen fibrils in the samples.

Rheometry and tensile experiments characterize the bulk properties of the hydrogel specimens, in shear and tension, respectively. Nanoindentation measures the local properties of the samples under local compressive indentation. The nanoindentation footprint in our experiment is on the order of 2 µm. This is in the range of the size of individual cells. Therefore, these local properties could be relevant to the microenvironment of cells, when they are cultured in this composite hydrogel.

It is widely accepted that the nanofibrous structure of collagen and its nano-topographic periodic pattern is important for cell attachment and growth [43,59,60]. As such, collagen has been used in tissue engineering applications [39,44,45,61,62]. However, fibrillar collagen in this form had not been previously reported for composite hydrogels. Collagen fibrils extracted from animal tissues are still fairly expensive. The high cost and concerns with immunogenicity associated with collagen need to be overcome before they are widely used for tissue engineering applications. Recent developments on Ovine collagen may resolve both issues, but this remains to be seen. Electrospun collagen nanofibers have been shown to be denatured collagen or simply gelatin. In general, the majority of electrospun nanofibers have a smooth topography and lack the characteristic nano-topography on the surface of the native collagen fibrils.

Although rheometry, tensile test [34], and AFM-nanoindentation [63,64] have been used for characterization of hydrogels, they are often used as a single tool for characterization. However, behavior of hydrogels in shear, tension and indentation could be different, as shown in our experimental data. Therefore, we have used all of these three techniques for characterization of the fabricated composite hydrogels. Native collagen fibrils have an elastic modulus on the order of several hundreds of kP to several GPa depending on their hydrated state [42]. Our results show that addition of an even small percentage of collagen to alginate hydrogel can result in improvement of the rheological and indentation properties of the hydrogel. Further effort is required to investigate the effect of adding higher concentration of collagen fibrils as well as to attempt to align the collagen fibrils in the hydrogel matrix to produce even stronger mechanical properties.

## 3. Experimental Section

Protonal^®^ LF10/60FT, Pharm-grade sodium alginate powder from FMC BioPolymer (Philadelphia, PA, USA) was used for preparation of the alginate hydrogel. Calcium Carbonate (CaCO_3_) was purchased from RICCA Chemical Co (Arlington, TX, USA). Calcium Sulfate Dihydrate (CaSO_4_·2H_2_O), Collagen from bovine Achilles tendon and GDL (d-(+)-Gloconic acid δ-lactone) were purchased from Sigma-Aldrich (St. Louis, MO, USA). Calcium Chloride Dihydrate (CaCl_2_·2H_2_O) was purchased from Fisher Scientific (Fair Lawn, NJ, USA). Sulfuric acid (H_2_SO_4_) was purchased from VWR (Radnor, PA, USA). Polydimethylsiloxane (PDMS) was purchased from Dow Corning Co. (Midland, MI, USA).

*Preparation of casting molds*: Molds with different geometries were designed to prepare samples for the tensile test, AFM nanoindentation, and rheometry experiments. For the tensile test, according to ASTM standard F2900-11 [65], dumbbell shaped geometry was used (Figure S4). For AFM nanoindentation and rheometry, disk shape mold was used (Figure S5). Several master molds with identical dimensions were machined from an Acrylic sheet. PDMS mix including the base materials and curing agent was poured into master molds with negative profile and allowed to cure in an oven below 80 °C for 2 h. After curing, the PDMS was peeled off the master mold. These PDMS molds were used as molds for casting hydrogel specimens for mechanical characterization (Figure S4B).

*Preparation of native collagen fibrils*: A stock solution of collagen fibrils was prepared by soaking ~500 mg collagen flakes in ~200 mL of 0.01 M sulfuric acid overnight. Subsequently, the solution was mixed using a blender for one hour to break down the collagen flakes to individual fibrils. The pH of the solution was adjusted to 6.5 by substituting the original solvent with DI water. The prepared collagen solution was left in the fridge so that the larger collagen bundles from the mixing process settled down. The supernatant solution, that contained mostly individual collagen fibrils, was separated and used for the preparation of the hydrogel samples. This was confirmed by placing several droplets of the solution on a glass slide and observing under an optical microscope. Collagen concentration in the final stock solution was measured to be ~1 mg/mL, which was considered as 4X and represents the maximum concentration. To measure the collagen concentration in the final solution, four batches of 5 mL from final solution were air dried and the dried films were weighed. The average of these measurements is reported as collagen concentration of 4X stock solution. Lower collagen concentration solutions 3X, 2X and 1X (1X ≅ 0.25 mg/mL) were prepared by diluting the 4X solution with the proper amount of DI water.

*Preparation of alginate hydrogel*: Alginate solution was prepared by dissolving 2% *w*/*v* of LF 10/60FT in DI water. Then 3 mg/mL (30 mM) calcium carbonate (CaCO_3_) powder was added to the solution and the solution was stirred for two min. 60 mM (10.68 mg/mL) GDL was added to this solution while the solution was vortexed for two min. To maintain the pH of the solution near neutral value, the ratio of CaCO_3_ to GDL was kept 1:2 [59,60,66]. The crosslinking process was controlled by introducing GDL for the activation of calcium ions from the calcium carbonate. GDL gradually reduces the pH of the solution, which results in the release of calcium ions and crosslinking of the alginate monomers. After this step, the prepared solution was immediately poured into the PDMS molds. The molds were kept in a humidity box for 24 h for the hydrogel to completely crosslink. To ensure the data are repeatable, at least five samples were prepared and tested for each experiment. The composite hydrogel samples were prepared by adding alginate to collagen solutions with different concentrations.

*Freeze-drying and SEM imaging*: Several of the hydrogel specimens were flash-frozen with liquid nitrogen, and then freeze-dried using a FreeZone freeze dryer system (LABCONCO, Kansas City, MO, USA), Figure S6. A ~15 nm gold film was sputter-coated onto the surface of the freeze dried samples prior to imaging with SEM. SEM images were acquired using a Zeiss-LEO Model 1530 variable pressure SEM (Zeiss, Oberkochen, Germany).

*AFM imaging*: AFM images of air-dried collagen solution and freeze-dried samples were obtained in air using MFP-3D-Bio (Asylum Research, CA, USA) with a cantilever “HQ:NSC15/Al-BS” (µMesch) with 40 nN/nm stiffness in AC mode (tapping mode) with frequency of 0.5 Hz.

*Tensile test experiment*: Tensile test experiments were performed using an Instron 5969 machine (Norwood, MA, USA) equipped with a pneumatic gripper and a 500 N load cell. To reduce the punching effect of the pneumatic gripper on the hydrogel specimen, a gripper adaptor was designed and fabricated that provides the possibility of adjusting the gripping pressure (Figure S7). Inner surfaces of the gripper adapter were covered with cardboard to prevent sample sliding. Prior to the tensile test, the cross-section of each sample was measured with a digital caliper. The sample was loaded onto the gripper adapter with an adjusted gap. To avoid pretension on the hydrogel samples a cardboard was placed between two gripper adapter to keep them at a constant relative distance and function as a frame for the specimen prior to the test. The samples were kept inside a humidity box prior to experiments to avoid dehydration of the specimens. Each sample was loaded onto the pneumatic gripper and the frame cardboard was cut prior to initiation of the experiment. All tensile tests were performed with a quasi-static strain rate of 1%/s.

*AFM Nanoindentation experiments*: Nanoindentation experiments were performed on fully hydrated hydrogel samples submerged in DI water. To prevent movement and floatation of hydrogel samples under AFM, the samples were secured from the bottom side to the petri dish and were firmly held from the top side using a costume fixture (Figure S5B). For nanoindentation experiments, a soft triangular AFM cantilever with a spring constant of *k* ~0.32 N/m was used. The AFM probe tip had an integrated 2 µm silicon oxide (SiO_2_) spherical probe tip (sQube^®^). Nanoindentation experiments were performed using a MFP 3D Bio-AFM (Asylum research, Santa Barbara, CA, USA). Before the nanoindentation experiment, the deflection sensitivity of the AFM cantilever was calibrated on a stiff substrate (Si). Nanoindentation experiments were performed on different areas from 10 µm × 10 µm to 90 µm × 90 µm for a total of 100 indentation points. We examined different displacement rates from 500 nm/s to 3 µm/s, which showed similar results.

*Rheometry experiments*: A Discovery Hybrid Rheometer (DHR-3) (TA Instruments, New Castle, DE, USA) was used to perform parallel-plates rheological experiments. The samples were disk-shaped with a diameter of 25 mm and a height of 3 mm. The storage and loss moduli of the samples were obtained in the frequency range of 0.1–100 Hz, under 0.5 N (1 kPa) compressive force.

*Statistical Analysis*: Statistical analysis was performed using Origin (V8.0988; OriginLab Corp, MA, USA) to determine the statistical differences. For tensile test data and nanoindentation data, statistical comparisons were performed with one-way analysis of variance (One Way ANOVA). For rheological data, since we had collagen concentration and frequency changes, statistical comparison was performed with two-way analysis of variance (Two Way ANOVA). Statistical significance for all tests was set to be at a *p* value <0.05.

## 4. Conclusions

In summary, we fabricated composite alginate-type I collagen fibril hydrogels and characterized their mechanical properties using rheometry, tensile experiment, and AFM-spherical probe nanoindentation. The results show that addition of collagen has a pronounced effect on the rheological and indentation properties of the hydrogel, while tensile properties showed minimal changes. Nanoindentation properties improve by more than 100%. Rheological properties for 4X collagen concentration showed several times improvement over alginate hydrogel with no collagen fibrils.

## Supplementary Materials

Supplementary materials can be accessed at: http://www.mdpi.com/1996-1944/8/2/0799/s1.
