# Supplementary material for: Alginate-Collagen Fibril Composite Hydrogel
Source: Materials (Basel). 2015 Feb 16;8(2):799–814. doi: 10.3390/ma8020799 (PMC5455273; doi:10.3390/ma8020799)
Supplement: Supplementary file 1 [file materials-08-00799-s001.pdf]

Supplementary Information

Table S1. Detailed tensile properties of the tested samples.

Collagen Concentration	*E* (kPa)	Failure Strain (%)	Failure Stress (kPa)	Toughness (J/m^3 × 10^3)
0X	133.17 ± 26.35	45.85 ± 9.59	46.72 ± 15.77	9.28 ± 5.35
1X	148.24 ± 26.37	47.68 ± 11.71	50.28 ± 10.54	10.50 ± 5.41
2X	159.72 ± 12.76	47.03 ± 12.08	53.91 ± 20.98	11.12 ± 7.86
3X	164.90 ± 39.80	46.55 ± 9.53	53.80 ± 13.55	11.55 ± 5.69
4X	165.10 ± 11.91	48.07 ± 9.31	61.20 ± 13.68	12.77 ± 5.15

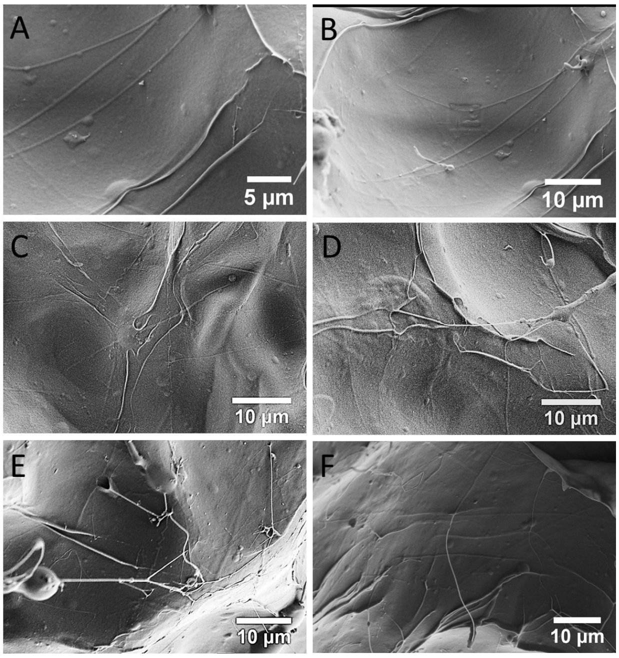

Figure S1. SEM images of composite hydrogel with collagen fibrils apparent on the surface of the hydrogel. (**A**) 1X; (**B**) 2X; (**C**) 3X; (**D–F**) 4X, respectively.

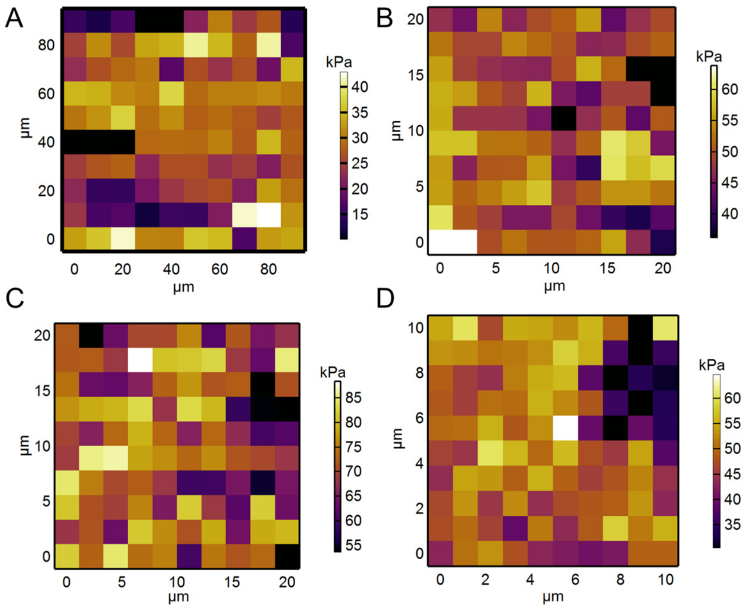

Figure S2. Representative nanoindentation maps on hydrogel samples with different concentration of collagen fibril. (**A–D**), 0X, 2X, 4X and 4X, respectively.

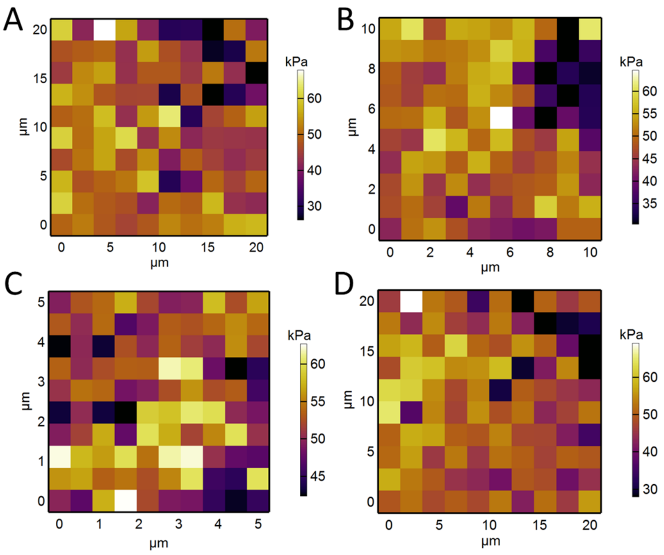

Figure S3. Representative nanoindentation maps with different indentation rates on hydrogel samples with 4X collagen fiber concentration. (**A**) 500 nm/s; (**B**) 1 μm/s; (**C**,**D**) 3 μm/s.

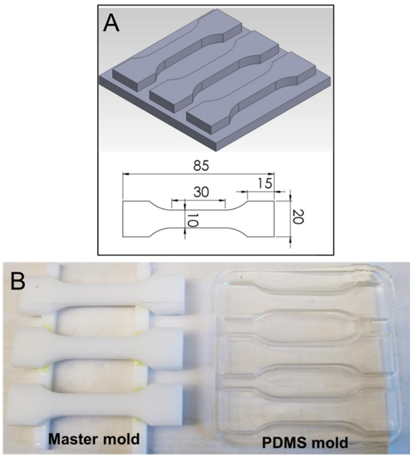

Figure S4. (**A**) 3D CAD model of the designed molds for tensile experiments; (**B**) Optical images of the machined master molds (left) and PDMS molds (right) for casting hydrogel specimens for tensile test.

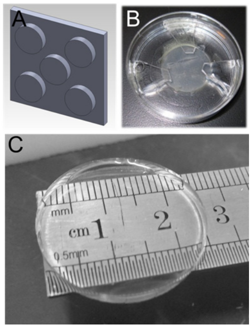

Figure S5. (**A**) 3D CAD model of the mold for casting of hydrogel samples for nanoindentation and rheology experiments; (**B**) An optical image of hydrogel samples prepared in special sample holder for AFM nanoindentation experiment; (**C**) An optical image of a casted hydrogel sample for nanoindentation and rheology experiments.

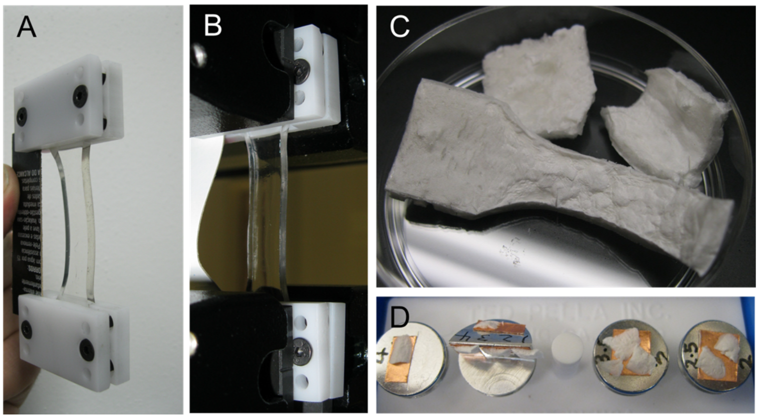

Figure S6. (**A**) A dog-bone hydrogel sample fixed between two gripper adapters; (**B**) A hydrogel specimen on the tensile machine, before experiment; (**C**) An optical image of a freeze-dried dog-bone hydrogel sample; (**D**) The freeze-dried samples on SEM sample holders for SEM imaging.

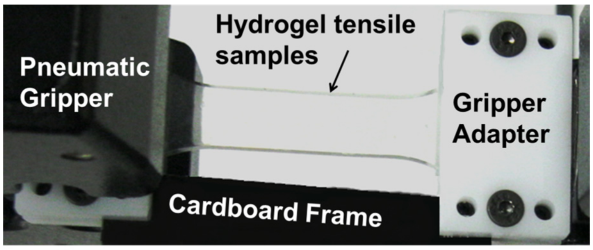

Figure S7. An optical image of a typical tensile test specimen. The gripper adapter is gripped by the pneumatic gripper of the tensile equipment. This setup reduces the pinching effect of the pneumatic grip on the hydrogel sample. The cardboard frame protects the soft hydrogel samples from over-stretching or bending during handling. Prior to initiation of the tensile experiment, the cardboard frame was cut using scissors.
